# Assessment of pain among a group of Nigerian dental patients

**DOI:** 10.1186/s13104-015-1226-5

**Published:** 2015-06-19

**Authors:** Emeka Danielson Odai, Adebola Oluyemisi Ehizele, Joan Emien Enabulele

**Affiliations:** Department of Oral and Maxillofacial Surgery and Pathology, University of Benin, Benin City, Nigeria; Department of Periodontics, University of Benin, Benin City, Nigeria; Department of Restorative Dentistry, University of Benin, Benin City, Nigeria

**Keywords:** Orofacial pain, Assessment, Dental patient, Visual analogue scale, Full cup test

## Abstract

**Background:**

Pain is considered a key symptom associated with possible impairment of oral-health-related quality of life and its assessment is important for the planning and evaluation of preventive and treatment effort. The tools for assessing pain must therefore be valid and consistent. The objective of this study was to assess dental patients’ level of pain based on the clinical diagnosis of their dental condition and the correlation between two pain assessment scales, Visual analogue scale (VAS) and the Full Cup Test (FCT), for the assessment of pain among dental patients.

**Methods:**

A total of 185 patients presenting at the University of Benin Teaching Hospital dental outpatient clinics with various forms of orofacial pain were included in this study. The mean VAS scores and mean FCT scores for the different dental conditions were compared. Agreement between VAS and FCT was evaluated using the Intra-class correlation (ICC) coefficients and Cronbach alpha coefficient was also calculated to assess consistency of the two pain scales.

**Results:**

Majority i.e. 95.1, 96.2 and 100% who presented with acute pulpitis, acute apical periodontitis and pericoronitis respectively, presented with moderate to severe pain levels (p < 0.05). Only 25.9 and 4% who presented with chronic marginal gingivitis and chronic pulpitis respectively presented with no pain (p < 0.05). A large proportion (75%) of patients with no pain had single diagnosis while more than half (52.1%) of those who presented with severe pain had multiple diagnoses (p = 0.025). The mean VAS and FCT scores for acute pain were 6.1 ± 2.1 and 5.9 ± 2.4 respectively and for chronic pain 3.9 ± 2.7 and 3.7 ± 2.7 respectively (P = 0.001). The interclass correlation coefficient revealed that the mean VAS and FCT scores were statistically correlated and reliable with a Cronbach alpha coefficient of 0.85.

**Conclusion:**

It can be concluded that patients who presented with either acute or chronic dental conditions may experience moderate to severe level of pain, with patients with multiple diagnoses experiencing more severe pain, and there is a correlation between the VAS and FCT for pain assessment among dental patients.

## Background

Orofacial pain is associated with significant morbidity and high levels of health care utilization [1]. It limits food choices and the pleasures of eating, restrict social contact and alter daily routine [2]. Pain is considered a key symptom associated with possible impairment of oral-health-related quality of life [3, 4]. The prevalence of orofacial pain is population dependent and prevalence of 18.5–58.8% has been reported by various studies worldwide [5–9]. However, a prevalence of 34% was reported in our immediate locality, Benin City [10].

Pain assessment is important because pain is a subjective phenomenon that is present when the individual experiencing it says it is and the individual is the most reliable source of information about location, quality, intensity, onset, and relieving, precipitating or aggravating factors of pain [11–13]. Inadequate pain assessment, with resultant difficulties in management of pain has however been reported by many studies [14–18].

Self report of pain can serve a reliable primary source of information although an individual’s lack of pain expression does not necessarily mean absence of pain [19]. It is considered a gold standard by which other assessment techniques may be judged, despite its limitations and biases and other assessment tools are necessary only in individuals who cannot self-report, such as the very young and those with cognitive impairment [20, 21].

Pain assessment tools used for self report can either be unidirectional or multidirectional, although the unidirectional ones are easier to use. Examples of unidirectional pain assessment tools are Visual analogue scales (VAS). Verbal rating scales (VRS), Graphic rating scales, Numerical rating scales, Verbal descriptor scales, Body diagrams, Computer graphic scales, Picture scales and Coin scales [22–24]. A more recent pain assessment scale called “Full Cup Test” (FCT) has also been suggested for pain evaluation, especially in patients with low education i.e. patients with informal education or with primary level of education [25, 26]. A Nigerian study reported correlation between VAS and FCT among dental patients undergoing tooth extraction [27].

The knowledge of patient’s level of pain is important for the planning and evaluation of preventive and treatment effort and this involves the point assessment of pain intensity at presentation. The objective of this study was therefore to determine the relationship between dental patients’ level of pain at presentation and the clinical diagnosis of their dental condition and also to determine the correlation between VAS and FCT for pain assessment among dental patients presenting with various forms of orofacial pain.

## Methods

A total of 185 patients presenting with various forms of orofacial pain at the University of Benin Teaching Hospital, Benin City were included in this cross-sectional study. Written informed consent was obtained from the study participants and approval was obtained from the Ethics and Research Committee of the college of medical sciences, University of Benin (REF no: CMS/PO/109/Vol.1/1115) before the commencement of the study in July, 2013.

Data collected include the patients’ demographic details, and past dental visit. Diagnosis of each case was made after proper history taking, clinical examination and radiographic investigation by qualified clinicians based on universally acceptable parameters. Pain assessment was done by each patient using VAS and FCT.

VAS is a uni-dimensional scale that is very useful in measuring pain intensity [28, 29]. It has the advantage of the easy and rapid application as well as low cost. It is line 10 cm in length with each end anchored by extreme descriptive (i.e. no pain vs the most severe pain). The patients were asked to mark their pain degree on the line between ‘no pain’ and ‘the most severe pain’. The place of the mark was then measured in centimeters [26]. VAS score 0 cm was categorized as no pain, 1–3 cm mild pain, 4–6 cm moderate pain and 7–10 cm severe pain.

For the FCT, a drawing of a cup was used. The patients were told ‘this cup is completely empty when there is no pain and completely full when your pain is the most severe. And now, how much of this cup is filled by your pain?’ The patients then drew a line on the cup to indicate the level of pain [26].

FCT score was calculated as follows: $\frac{\text{Height of line}}{\text{Height of cup}}\; \times \; 100$. Maximum score (100%) on FCT was given a value of 10 for easy Comparism with VAS.

The collected data was analyzed using the statistical package for social sciences (SPSS) version 15.0. VAS scores were cross tabulated against patients’ age, gender and the number of diagnosis. Chi-square test was used to determine statistical significance. The level of significance was set at P value <0.05. For the purpose of comparing the two scales, the various diagnoses were broadly grouped into acute pain, chronic pain and conditions not pain related. The mean pain scores for the different groups were compared using analysis of variance (ANOVA) and the Tukey’s Post Hoc test was done for multiple comparisons. Agreement between VAS and FCT was evaluated using the Intra-class correlation (ICC) coefficients computed at 95% confidence interval. Cronbach alpha coefficient was also calculated to assess consistency of the two pain scales.

## Results

More of the study participants (54.6%) were in the 20–39 age group, females (54.1%) and had attained a tertiary level of education (56.8%). More than half of the participants (54.1%) had single diagnosis and 53% had no previous dental visit (Table 1).

**Table 1:** Demographic characteristics of study participants

Characteristics	n (%)
Age group (years)	
18–20	20 (10.8)
20–39	101 (54.6)
40–59	48 (26.0)
60 and above	16 (8.6)
Gender	
Male	85 (45.9)
Female	100 (54.1)
Highest level of education	
Informal	5 (2.7)
Primary	26 (14.1)
Secondary	49 (26.5)
Tertiary	105 (56.8)
Type of diagnosis	
Single diagnosis	100 (54.1)
Multiple diagnosis	85 (45.9)
Total	185 (100.0)

**Figure 1:**
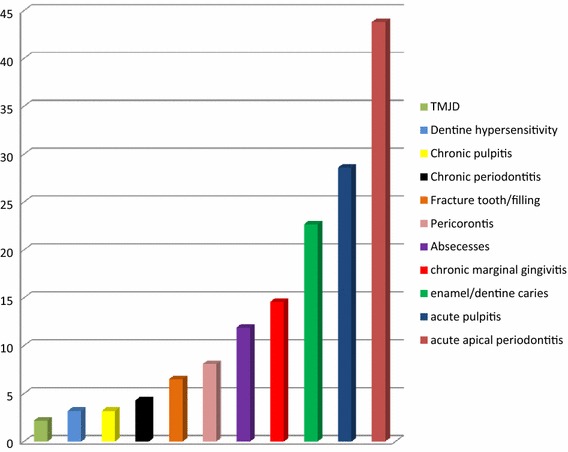
The participants’ diagnosis.

The following were 10 most frequently diagnosed conditions among the participants; Acute apical periodontitis (43.8%), acute pulpitis (28.6%), enamel/dentinal caries (22.7%), chronic marginal gingivitis (14.6%), chronic pulpitis (13.5%), abscesses (11.9%), pericoronitis (8.1%), fractured teeth/fillings (6.5%), chronic periodontitis (4.3%), and dentinal hypersensitivity (3.2%) (Figure 1).

Majority i.e. 95.1, 96.2 and 100% who presented with acute pulpitis, acute apical periodontitis and pericoronitis respectively, presented with moderate to severe pain levels (p < 0.05). Only 25.9 and 4% who presented with chronic marginal gingivitis and chronic pulpitis respectively presented with no pain (p < 0.05) (Table 2).

**Table 2:** Relationship between clinical diagnosis and pain scores using VAS

Diagnosis	VAS Grading				Total, n (%)	X^2^	P value
	No pain, n (%)	Mild, n (%)	Moderate, n (%)	Severe, n (%)			
Acute pulpitis	0 (0.0)	2 (3.8)	28 (52.8)	23 (43.4)	53 (100.0)	10.45	0.015
Acute apical pericoronitis	0 (0.0)	4 (4.9)	40 (49.4)	37 (45.7)	81 (100.0)	17.60	0.001
Pericoronitis	0 (0.0)	0 (0.0)	10 (66.7)	5 (33.3)	15 (100.0)	7.40	0.060
Chronic marginal gingivitis	7 (25.9)	3 (11.1)	11 (40.7)	6 (22.2)	27 (100.0)	14.76	0.002
Chronic pulpitis	1 (4.0)	7 (28.0)	6 (24.0	11 (44.0)	25 (100.0)	10.16	0.017

A large proportion (75%) of patients with no pain had single diagnosis while more than half (52.1%) of those who presented with severe pain had multiple diagnoses (p = 0.025) (Table 3).

**Table 3:** Relationship between the number of clinical diagnosis and pain scores using VAS

Number of diagnosis	VAS grading				Total, n (%)	X^2^	P value
	No pain, n (%)	Mild, n (%)	Moderate, n (%)	Severe, n (%)			
Single	9 (75.0)	15 (71.4)	42 (51.9)	34 (47.9)	100 (54.1)	5.01	0.025
Multiple	3 (25.0)	6 (28.6)	39 (48.1)	37 (52.1)	85 (45.9)		
Total	12 (6.5)	21 (11.4)	81 (43.8)	71 (38.4)	185 (100.0)		

The mean VAS and FCT scores for acute pain were 6.1 ± 2.1 and 5.9 ± 2.4 respectively and for chronic pain 3.9 ± 2.7 and 3.7 ± 2.7 respectively (P = 0.001). Analysis of Variance revealed that there is a statistically significant difference in the means scores for the different types of pain when the two scales were used for pain estimation (Table 4).

**Table 4:** Comparing the mean pain score for the different types of pain using ANOVA

	Mean	Std deviation	Std error	F	Sig
VAS					
Acute pain	6.11	2.14	0.18	29.92	0.001
Chronic pain	3.92	2.71	0.55		
Not pain related	2.06	2.89	0.70		
FCT					
Acute pain	5.90	2.37	0.20	15.51	0.001
Chronic pain	3.75	2.69	0.55		
Not pain related	2.98	3.62	0.88		

The Post Hoc test, on the mean pain scores from the VAS, revealed a statistically significant difference between the mean scores for the different types of orofacial pain (acute and chronic pain as well as non pain related cases). There was however no statistically significant difference between mean pain scores for chronic pain and non pain related cases from the FCT (Table 5).

**Table 5:** Multiple comparison of mean pain score for different types of pain using Tukey’s Post Hoc test

	Mean difference	Std error of mean	Sig
VAS			
Acute pain vs chronic pain	2.19	0.51	0.001
Acute pain vs not pain related	4.05	0.59	0.001
Chronic pain vs acute pain	−2.19	0.51	0.001
Chronic pain vs not pain related	1.86	0.73	0.031
Not pain related vs acute pain	−4.05	0.59	0.001
Not pain related vs chronic pain	−1.86	0.73	0.031
FCT			
Acute pain vs chronic pain	2.15	0.56	0.001
Acute pain vs not pain related	2.93	0.65	0.001
Chronic pain vs acute pain	−2.15	0.56	0.001
Chronic pain vs not pain related	0.77	0.81	0.604
Not pain related vs acute pain	−2.93	0.65	0.001
Not pain related vs chronic pain	−0.77	0.81	0.604

The interclass correlation coefficient revealed that the mean VAS and FCT scores were statistically correlated and reliable with a Cronbach alpha coefficient of 0.85.

## Discussion

Health professionals tend to under or overestimate their patients’ level of pain or assume that some conditions should be painful while some should be painless. The result of this study reveals individuals with same diagnosis have varying level of pain and that no dental condition is painless in all cases. This supports the previous report that the extent and quality of the damage, the individual’s previous experience of pain and his emotional state at the time all determine the individual’s level of pain [30]. There is therefore a need to assess each case.

According to Dionne et al., pain assessment tools should be understandable, clinically relevant, closely related to the response of the patient, responsive to change, valid in a variety of pain conditions and its clinical utility should be demonstrable [31]. Although VAS is highly useful, especially when the main objective is not to assess the multi-dimensional nature of pain, there have been attempts to have alternatives that will be easily understandable by the younger age group. It was reported that children have more difficulty understanding the use of VAS when compared to a more graphic pain assessment scale like Wong–Baker faces pain rating scale (WBFPS) [32].

The VAS has also been reported to have more practical difficulties than the Verbal rating scale (VRS) and numeric rate scale (NRS) and for simplicity, patients prefer the VRS [33]. It was however documented that VRS lacks sensitivity and the data it produces can be misunderstood [22]. A simple tool that can be easily understandable by even persons with low education was therefore highly needed. The full cup test was found useful for assessing pain in patients with low education because it does not need any numerical or word skills, and is easy to understand and to complete [26].

Previous studies revealed that the mean VAS and FCT scores did not differ significantly and were highly correlated and reliable [27, 31, 34]. The result of this study agrees with these previous studies because of the high correlation coefficient score recorded. The findings of this study show that VAS was able to show difference in pain score between acute, chronic and non pain related cases. FCT on the other hand showed difference in pain scores from acute and chronic cases but the distinction between pain scores of chronic cases and non pain related cases was not so obvious. This result is different from a result from a previous study carried out in Turkey, among persons with low education, where it was concluded that FCT is useful for both assessing and differentiating changes in pain.

Generally, chronic conditions are associated with dull pain or no pain at all but it is highly unpredictable what an individual will present with. The level of pain will depend on whether at the time of presentation there is an acute exacerbation of the chronic condition or not. It will also depend on the individual’s pain threshold. This individual variation may have been the reason for the pattern observed in this study. It is therefore recommended that FCT should be used to assess pain in larger groups of patients with various types of orofacial pain, at the time of presentation and after the management of the condition they presented with, to ascertain its usefulness for detecting changes in pain levels. Also since most of the pain assessed in this study can be classified as dental oral pain, future studies should include other “non dental” oral pain, headaches and facial pain.

## Conclusion

It can be concluded that patients who presented with either acute or chronic dental conditions may experience moderate to severe level of pain and that patients with multiple diagnoses present with more severe pain. Also, it can be concluded that there is a correlation between the VAS and FCT for pain assessment among dental patients but the type of orofacial pain measured has an effect on the consistency of the two pain assessment scales.

## Abbreviations

VAS: Visual analogue scale
FCT: Full Cup Test
